# Newton’s cradle-like allosteric mechanism explains regulatory RsmE RNA binding

**DOI:** 10.21203/rs.3.rs-6227789/v1

**Published:** 2025-04-15

**Authors:** Esteban Finol, Fred F. Damberger, Miroslav Krepl, Timo Flügel, Priscilla Dietrich, Thomas C.T. Michaels, Beat Vögeli, Jiří Šponer, Frédéric H-T. Allain

**Affiliations:** 1 Institute for Biochemistry, Department of Biology, ETH Zurich, 8093, Zurich, Switzerland; 2 Institute of Biophysics of the Czech Academy of Sciences, Kralovopolska 135, 61200, Brno, Czech Republic; 3 Department of Biochemistry and Molecular Genetics, University of Colorado Anschutz Medical Campus, Aurora, CO 80045, USA.

## Abstract

In the bacterial Csr/Rsm system, non-coding RNAs activate mRNA translation by removing homodimeric Csr/Rsm proteins from the ribosome-binding sites of mRNAs. In *Pseudomonas protegens*, each RsmZ ncRNA sequesters up to five RsmE dimers sequentially and specifically within a narrow affinity range, functioning as a ‘protein sponge’. Although the RsmE binding cascade is cooperative, binding of the highest affinity stem-loop RNA in RsmZ (SL2) reduces RNA binding affinity at the second site by 10- to 30-fold. This unusual negative cooperativity may facilitate RsmE release from tightly bound mRNA for handover to the non-coding RNA, yet the underlying mechanisms remain unclear. Using Isothermal Titration Calorimetry, NMR spectroscopy and Molecular Dynamic simulations, we show that the initial binding event increases conformational entropy at the empty site, partially unfolding the C-terminal helix. Moreover, we reveal an allosteric mechanism coupling RNA binding at the first site to conformational changes at the second site, explaining the reduced affinity of the second binding event. The anti-parallel β-sheets in the RsmE dimer facilitate communication between sites, with H-bond constriction at the bound site and relaxation at the empty site, resembling a Newton’s cradle.

In Gammaproteobacteria such as *Salmonella*, *Pseudomonas*, *Escherichia*, *Erwinia,* and *Vibrio*, homodimeric proteins from the carbon storage regulator/repressor of secondary metabolites (Csr/Rsm) family bind to the Shine-Dalgarno (SD) sequence in the 5’ untranslated region of messenger RNA (mRNA). As an intertwined dimer, they employ one of their two composed RNA binding sites to recognize the Guanine-Guanine-Adenine (GGA) motif in the SD sequence, while the other binding site engages a second GGA motif in the mRNA^1^. Their high affinity for the SD allows Csr/Rsm protein dimers to compete with the small (30S) subunit of the ribosome, thereby repressing translation initiation of hundreds of mRNAs^2^. Consequently, Csr/Rsm proteins serve a global regulatory system of gene expression for many fundamental cellular processes. They repress biofilm formation, quorum sensing, gluconeogenesis and glycogen metabolism, and they activate virulence, glycolysis, cell motility and pathogenesis^3,4^.

In response to environmental stimuli, these bacteria express small non-coding RNA (ncRNA) that can sequester and remove Csr/Rsm-type proteins from mRNA, allowing for their translation^5^. In the *Pseudomonas protegens* CHA0 strain (formerly *P. fluorescens*), each 127-nucleotide RsmZ ncRNA molecule seizes four RsmE dimers, making use of eight GGA motifs: four located in the loops of stem-loop (SL) RNA and four in single-stranded regions^6^. In isolation, shorter RNA molecules containing the individual GGA motifs bind RsmE dimers with dissociation constants (K_D_) in the μM range^7^. The only exception is the GGA motif within the SL2 in RsmZ ncRNA, which binds RsmE at very high affinity (K_D_ in the low nM range). Since all the isolated binding sites have micromolar or lower dissociation constants, RsmZ sequesters RsmE dimers in a sequential and cooperative manner^8^. As shown previously^8^ and repeated here (Fig.1), titration of the RsmE dimer with isolated SL2-RNA exhibits a much reduced affinity for the second RNA binding event (10- to 30-fold, see Supplementary Table 1). This represents an example of anti-cooperativity. Moreover, examination of the backbone amide (NH) cross peaks of RsmE upon binding to only one SL2 RNA, revealed the presence of two signals from the singly bound (semi-*holo*) state whose shifts are distinct from both the unbound (*apo*) and the doubly bound (*holo*) form of the dimeric protein (Fig.1b). For each amide displaying these two signals, one could generally be identified with the bound site and the other with the empty site, and thus clearly reported on the broken symmetry due to the singly bound RsmE homodimer. Although the NH-CSPs (Chemical Shift Perturbations upon RNA binding) of the bound site were generally similar in magnitude to those of the *holo* dimer (Fig.1c), the distinguishable shift positions of the singly bound versus doubly bound states suggest that the strong RNA binding to the first binding site is different from the doubly bound *holo* state. In addition, shift changes at the empty site suggest that RNA binding to the first site allosterically alters the conformation of the second, empty RNA binding site. Here, we investigate the molecular basis of this negative RNA binding cooperativity to decipher its allosteric mechanism.

## The first SL2 RNA binding results in multiple conformations in the bound carboxy-terminal disordered region (CDTR)

In order to understand the origin of negative allostery, we examined in detail the NMR spectra of the RsmE dimer bound to one SL2 RNA. Close inspection of the ^1^H-^15^N-HSQC spectrum of the semi-*holo* form revealed a set of additional low-intensity backbone amide signals (Fig.2a), suggesting the existence of a minor conformation in slow exchange with the major form. In the ^1^H-^13^C-HSQC, we could even detect three conformations for some side chains (**Supplementary Fig.1a-c**). Quantifying the NMR signals in the ^1^H-^13^C HSQC spectrum indicated that the abundance of these distinguishable conformations in slow exchange is approximately 61%, 33% and 6%. In what follows we term these semi-*holo* states: state I (or major), II (or minor), and III respectively. From the assignments, the regions involved in slow conformational exchange among these states could be mapped to the α-helix and CTDR of the RNA-bound side of the semi-*holo* complex as exemplified by the NMR signals of the bound Isoleucine 47 and Isoleucine 51 in the α-helix and Alanine 57 in the CTDR respectively (B-I47, B-I51 and B-A57 in Fig.2b–c, **Supplementary Fig.1 and 2, Supplementary Text 1**). For clarity, we indicate bound site and empty site residues with B- and E-respectively. In the previously determined structure of the *holo* complex in solution^7^, amino acids in these regions interact with adenine 26 (Ade26) in the SL2 hexa-loop of RsmZ ncRNA (Fig.2b). The aromatic nature of the adenine base can induce a ring current effect on neighbouring nuclei. Thus, subtle changes in Ade26 base orientation can lead to distinct proton chemical shifts for protein sidechains in proximity. These sidechains in the major (state I) conformation of the semi-*holo* complex underwent similar CS perturbations upon SL2 binding to those observed in the *holo* complex (**Supplementary Fig.2**), confirming that this major conformation has a local structure closely similar to the *holo* structure. To probe the RNA-protein contacts in the three semi-*holo* states of SL2-RsmE dimer complex, a 2D f1-filtered, f2-^13^C edited NOESY experiment was recorded with ^13^C,^15^N-labeled protein and unlabelled SL2 RNA. Intermolecular NOEs for the major and minor states could be detected and they were associated with subtle chemical shift differences of Ade26 as well (H2^I^= 8.116 ppm and H2^II^= 8.121 ppm; H8^I^= 8.215 ppm and H8^II^= 8.221 ppm). Clear intermolecular NOEs between Ade26 H2 and H8 protons and the methyl protons of B-I51 Cδ1 in the bound α-helix for the major (I) and both minor conformations confirmed that these interactions persist even in the minor forms (Fig.2c). In contrast, no intermolecular NOEs were observed between Ade26 and the state II of B-A57 in the CTDR (e.g., no Ade26-B-A57^II^Hβ NOE in Fig.2c), suggesting the existence of a distinct CTDR conformation with no Ade26 contacts.

## MD simulations support the existence of a second bound state where the CTDR does not contact Ade26

To gain further spatial and temporal insights into the conformational space explored by the RsmE dimer in the presence of one SL2 RNA, we performed explicit solvent atomistic molecular dynamics (MD) simulations. For this purpose, one SL2 RNA was removed from the previously determined *holo* SL2-RsmE dimer structure, this semi-*holo* structure was then equilibrated and four 10-μs MD simulations were performed. To track structural changes in the Ade26-RsmE dimer interface, the distances between the Ade26 H atoms and the methyl group H atoms in the SL2-bound α-helix (B-I47Cγ2 and B-I51Cδ1) and CTDR (B-A57Cβ) of RsmE dimer were measured throughout these simulations. The two-dimensional plots correlating these distances (for instance, Ade26H2–B-I47Hγ2 and Ade26H2–B-A57Hβ distances in Fig.2d) showed two clear peaks of abundant conformations, suggesting that the CTDR can adopt a second conformation, where the Ade26H2–B-A57Hβ distance is greater than 10 Å, while the Ade26H2–B-A47Hγ2 distance remained short, indicating Ade26 still interacted with the bound α-helix of RsmE. To characterize this second conformation, 20 frames were retrieved from the MD simulations; ten frames from the most abundant peak which is similar to the *holo* structure, and ten frames from the second peak containing conformations where the CTDR is not interacting with Ade26 (Fig.2e). In this second conformation, the carbonyl of B-P58 interacts with the sidechain amide group of B-Q52 of the α-helix (Fig.2f). By tracking the B-Q52Hε-B-P58O atomic distance across the four 10-μs MD simulations (**Supplementary Fig.3a**), a single event was detected which showed the emergence of this second conformation, suggesting that the transition represents a rare event on a 10-μs timescale. Three additional 10-μs MD simulations that started from this minor conformation indicated that it is similarly stable on the μs MD timescale (**Supplementary Text 2, Supplementary Fig.3b**). Support that the minor state detected in the MD is related to the minor state identified by NMR is derived from large amide shift differences observed between major and minor forms for the sidechain of B-Q52 and for the backbone of B-T56 (**Supplementary Fig.4**). Collectively, these observations support the existence of at least two slowly exchanging conformations associated with the loss of Ade26-CTDR contacts in the semi-*holo* SL2-RsmE dimer state.

## Contacts between the RNA stem and the β3-β4 loops contribute to increase the first binding affinity.

Analysing other regions of the complex in the MD trajectories, we saw that the phosphate backbone of the stem of the SL2 RNA formed transient interactions with residues from the β3-β4 loop of both monomers (Fig.3a and **Supplementary Fig.5a**). We observed repeated close approaches (< 5 Å) between the E-Q28 Cα in the empty site β3-β4 loop and Cyt20P in the SL2-stem (**Supplementary Fig.5b**). These interactions are consistent with the experimentally observed NH-CS differences in the β3-β4 loops of RsmE dimer between the semi-*holo* and the *holo* states (**Supplementary Fig.6a-b**), as well as the CS perturbations in the side-chain amide of Q28 in the empty β3-β4 loop of the *semi-holo* state (**Supplementary Fig.6c**). These interactions could be mediated by sodium ion bridges, as suggested by high sodium occupancy at this contact throughout the MD simulations (**Supplementary Text 3, Supplementary Fig.5a**). We therefore investigated the role of electrostatic interactions in the SL2 RNA binding to RsmE dimer by performing SL2-RsmE dimer titrations using ITC with different NaCl concentrations in the buffer solution (Extended data 1). Increased ionic strength reduced the first binding affinity proportionally more than the second one, supporting the finding that sodium ions play a role in creating a difference between the two binding events.

To investigate the role of the RNA stem contacts with the protein in the anti-cooperativity, a series of experiments were performed using a truncated SL2 RNA with the stem region shortened to 4 base-pairs (4bpSL2 RNA), which modelling studies indicated would be unable to contact the β3-β4 loop of the RsmE empty site. The ITC of the 4bpSL2 RNA binding to the RsmE dimer showed that the affinity is substantially reduced for the first binding event, with a remarkable reduction of the enthalpic (−30.15%) and entropic (−34.74%) components, as compared to the second binding event (−12.93% and −16.85%, respectively) (Fig.3b–c). Strikingly, the negative cooperativity observed for the cognate SL2 with a ratio of K_D2_/K_D1_ of ~20 is reduced to only ~3. Moreover, the semi-*holo* 4bpSL2-RsmE dimer complex showed slightly reduced NH-CS perturbations as compared to the NH-CS perturbation in the semi-*holo* SL2-RsmE dimer complex, including the backbone and side chain amides in the empty site’s β3-β4 loop (Fig.3d and **Supplementary Fig.7**, respectively). Interestingly, the semi-*holo* 4bpSL2-RsmE dimer complex lacked the multiple conformational states that were observed in the semi-*holo* SL2-RsmE dimer complex (Fig.3e–f). These findings indicated that the MD-observed transient contacts between the SL2 RNA stem and the RsmE dimer may increase the binding affinity of the first binding event and showed that the additional semi-*holo* states were dependent on the presence of an extended RNA stem.

In summary, the gain in overall free energy of SL2 RNA binding, relative to 4bpSL2, may come from an entropic gain provided by the multiple conformations involving Ade26 in the semi*-holo* complex as well as enthalpic gains due to an interaction of the RNA stem with the β3−β4 loops of both monomers. Yet, the reduced binding affinity persisted for the second 4bpSL2 RNA binding event with about a 3-fold reduction in affinity on shortening the stem of SL2. (Fig.3b). This prompted us to further explore the structural and dynamics changes at the empty binding site.

## RNA binding causes increased dynamics at the empty binding site

Three observations for the semi-*holo* SL2-RsmE complex revealed increased dynamics in its empty binding site compared to the *apo* state. First, additional line-broadening of backbone amide peaks, indicative of motions on the μs-ms timescale, was observed in the ^1^H^15^N-HSQC spectrum for residues in the β1 and β2 strands, the β4-β5 loop and the α-helix of the empty binding site, as well as for some residues in the β3 strand, which links the two binding sites (Fig.4a and **Supplementary Fig.8**). Second, quantitative analysis of Cα and Cβ CS indicated that some residues in the empty α-helix were even less ordered in the semi-*holo* complex than in the *apo* state, suggesting a shift in its folded-unfolded equilibrium. (**Supplementary Text 4, Supplementary Fig.9**). Consistent with this partial unfolding, the empty site α-helix also appeared to lose contacts with the β-sheet of the RsmE dimer (**Supplementary Fig.10**). Third, ^15^N{^1^H}-heteronuclear NOE^9^ experiments showed that binding of one SL2 RNA led to increased sub-nanosecond timescale dynamics of the β1, β2 and especially the β5 strand and the α-helix of the empty binding site, as compared to the *apo* state (Fig.4b), whereas much of these additional dynamics are quenched upon binding of the second SL2 RNA (Fig.4c). Interestingly, additional fast timescale backbone motion was also detected in some residues of the bound β3 and β4 strands. Since the β3 strands reside in the middle of the RsmE β-sheet, we wondered if these central β-strands serve to couple the two binding sites allosterically. That is, binding of one SL2 RNA may disrupt the rigidity of the empty binding site and contribute to destabilizing the empty site α-helix.

To test this hypothesis, the SL2-induced changes in the β-sheets of the RsmE dimer were investigated. Considering that the backbone amides serve as donors in the extensive network of hydrogen bonds (H-bonds) that stabilizes these β-sheets, we analysed their temperature dependence to provide information about their strength. The NH-CS are known to shift upfield (lower chemical shifts) linearly with increasing temperatures, resulting in a negative slope, which defines the so-called temperature coefficient (Tc)^10^. For hydrogen-bonded amides, subtle CS changes arise from thermal expansion of the H-bonds length, leading to weakly negative or even flat slopes. Conversely, the non-hydrogen-bonded amides show steeper negative slopes due to their higher solvent exposure and faster chemical exchange rate^11^. Furthermore, non-linearity in ^1^H-CS temperature dependence may indicate the presence of alternative conformations. Non-linearity was indeed observed for a third of the backbone amide protons in the *apo* RsmE dimer (**Supplementary Fig.11a**). This implies that the RsmE dimer can undergo a temperature-dependent conformational change, with a transition between 303K and 308K. To estimate the magnitude of these structural changes, we calculated the difference between the Tc slopes before and after the transition for every backbone amide (**Supplementary Fig.11b**) and mapped the amide protons that underwent the temperature-dependent transition onto the known structure of the RsmE dimer (Fig.5a). They clustered in the β2, β3 and β5 strands and the α-helix of the *apo* RsmE dimer. The largest changes corresponded to the hydrogen-bonded amide protons that stabilize the intra-monomeric β2-β3 and inter-monomeric β2-β5 interactions. This analysis of the *apo* RsmE dimer revealed that the β-sheets and the α-helices can undergo coordinated structural changes and access additional states in a temperature-dependent manner.

We next analysed the semi-*holo* SL2-RsmE dimer complex which also showed multiple residues with a temperature-dependent transition. To assess whether the binding of one SL2 RNA tightened or loosened the H-bonds in the β-sheets of the RsmE dimer, the changes of the Tc slopes between the *apo* and semi-*holo* states were measured (**Supplementary Fig.12**). Mapping of these changes onto the known structure of the RsmE dimer revealed two clusters of SL2-induced Tc changes (Fig.5b). One cluster involved the tightening of H-bonds that stabilize the β1-β4, β3-β4, β2-β5 interactions and the α-helix in the bound site, as well some H-bonds that connect the bound β2 strand with β3 in the empty site. In a second cluster, we observed, on the contrary, a weakening of the H-bonds in the β1-β4 and β2-β5 interactions and in the α-helix of the empty binding site. These observations are further supported independently by Hydrogen-Deuterium exchange (HDX) rate changes of backbone amides in the *apo* and semi-*holo* RsmE dimer (Fig.5c, **Supplementary Fig.13**). The binding of one SL2 RNA reduced the HDX rate in several backbone amides in the bound site while increasing HDX rates in the empty binding site. These two independent methods strongly suggest that the binding of one SL2 RNA triggers a concerted structural and dynamic change in the RsmE dimer, whereby the dynamics of the bound binding site is quenched, resulting in increasing dynamics at the empty binding site leading to a partial unfolding of the α-helix at the empty site. The backbone hydrogen-bonding network of two anti-parallel beta-sheets serves as a conduit to connect the two binding sites. The higher conformational entropy associated with increased dynamics at the empty site of the semi-*holo* complex can compensate for the reduced entropy of the binding site, thereby favourably contributing to the free energy of binding. In turn, a second RNA binding in the yet empty site is therefore weakened since a larger entropy change is needed to achieve RNA binding, furnishing an explanation for the observed anti-cooperativity.

## Discussion

**Figure 1:**
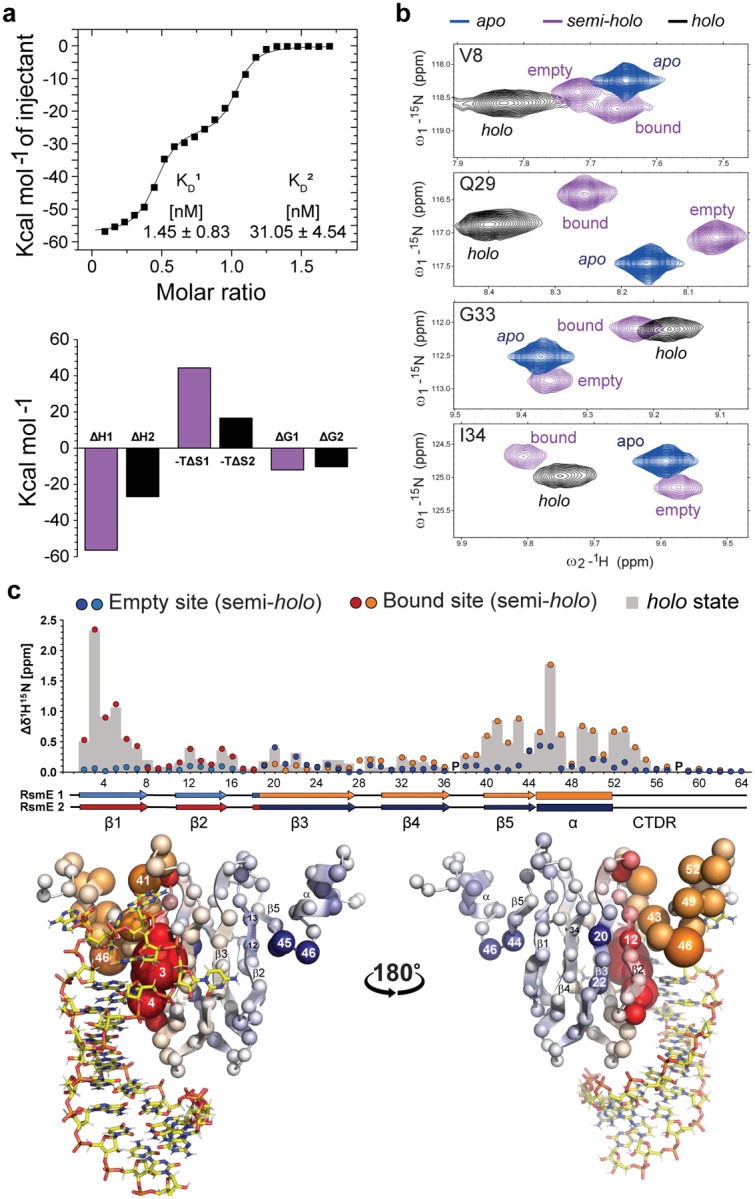
Negative allostery with two distinct intermediate states in RsmE dimer. **a**, Top panel shows Isothermal Titration Calorimetry (ITC)-derived binding curve of SL2 RNA titrated to the RsmE dimer. The molar ratio corresponds to the RNA - RsmE monomeric ratio. Bottom panel shows a bar-plot with thermodynamic changes for the first and second binding events. Values can be found in Supplementary Table 1. **b**, Four isolated backbone amide peaks in the overlaid ^1^H^15^N-HSQC spectra of the *apo* (blue), semi-*holo* (purple) and *holo* (black) SL2-^15^N-RsmE dimer states. **c**, NH-CSPs in the RsmE dimer upon binding of one SL2 RNA. Top panel, NH-CSPs in the bound and empty sites. Considering that the binding sites are composed of residues from both RsmE monomers, the dots for the NH-CS in the semi-*holo* state are differently colour-coded for the two monomers (light and dark colours) and the two binding sites (blue and red/orange). The underlying grey bar plot corresponds to NH-CSPs upon binding of two SL2 RNAs. The letter P indicates proline residues. Bottom panel, structural model of the RsmE dimer bound to one SL2 RNA with NH-CSPs observed at the bound and empty sites indicated by coloured spheres. Residues are colour-coded according to the secondary structure diagram below the NH-CS plots in *c*. Intensity of colours and diameters of spheres indicate the magnitude of NH-CSPs at every residue. The structure model for the semi-*holo* complex is derived from the *holo* SL2-RsmE dimer complex (PDB:2MFE), from which one SL2 RNA was removed.

**Figure 2:**
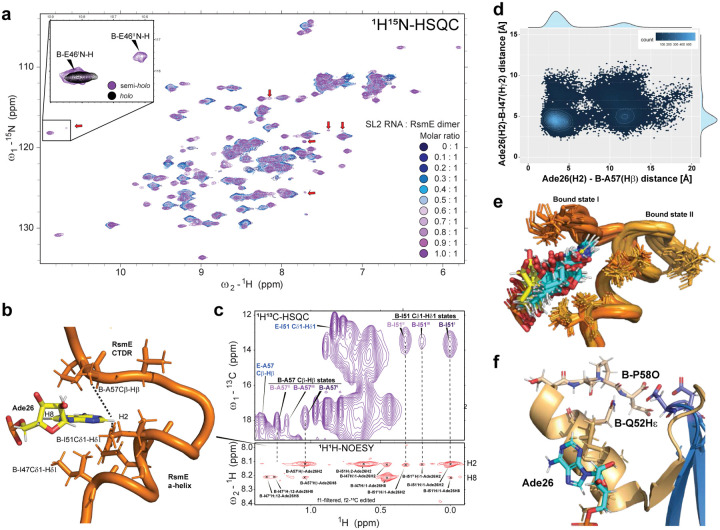
The binding of one SL2 RNA leads to multiple slowly exchanging conformations in the bound α-helix and CTDR of RsmE dimer. **a**, Overlaid ^1^H^15^N-HSQC spectra from the titration of SL2 RNA into the ^15^N-RsmE dimer, where only the first binding site was saturated. The insert shows an expanded view of the NH peak from bound Glutamate 46 (B-E46) in the ^1^H^15^N-HSQC spectra from semi-*holo* (purple) and *holo* (black) ^15^N-RsmE dimer. The B-E46 state II NH peak (B-E46^II^) is also labelled. **b**, α-helix and CTDR of the RsmE dimer bound to Ade26, as previously determined in solution (PDB: 2MFE). **c**, top spectrum: methyl region in the ^1^H^13^C-HSQC spectrum of the semi-*holo* RsmE dimer. Labels indicate the CS of I51Cδ1-Hδ1 and A57Cβ-Hβ in the empty site (blue), minor states II and III (light purple and purple) and major bound (dark purple) conformations. Bottom: Ade26 H2 and H8 NOE towers in the 2D f1-filtered, f2-^13^C edited NOESY spectrum. **d**, 2D-plot of correlated atomistic distances in the 10 μS MD simulations (total: 400000 frames). **e**, Structural alignment for 10 representative snapshots of the two abundant MD-derived conformations in the α-helix and CTDR of the semi-*holo* RsmE dimer (State I and II are coloured in dark orange and orange, respectively). Ade26 is shown as coloured sticks (carbons are yellow for state I and cyan for state II). **f**, Contacts observed in the bound state II conformation in MD simulations.

**Figure 3:**
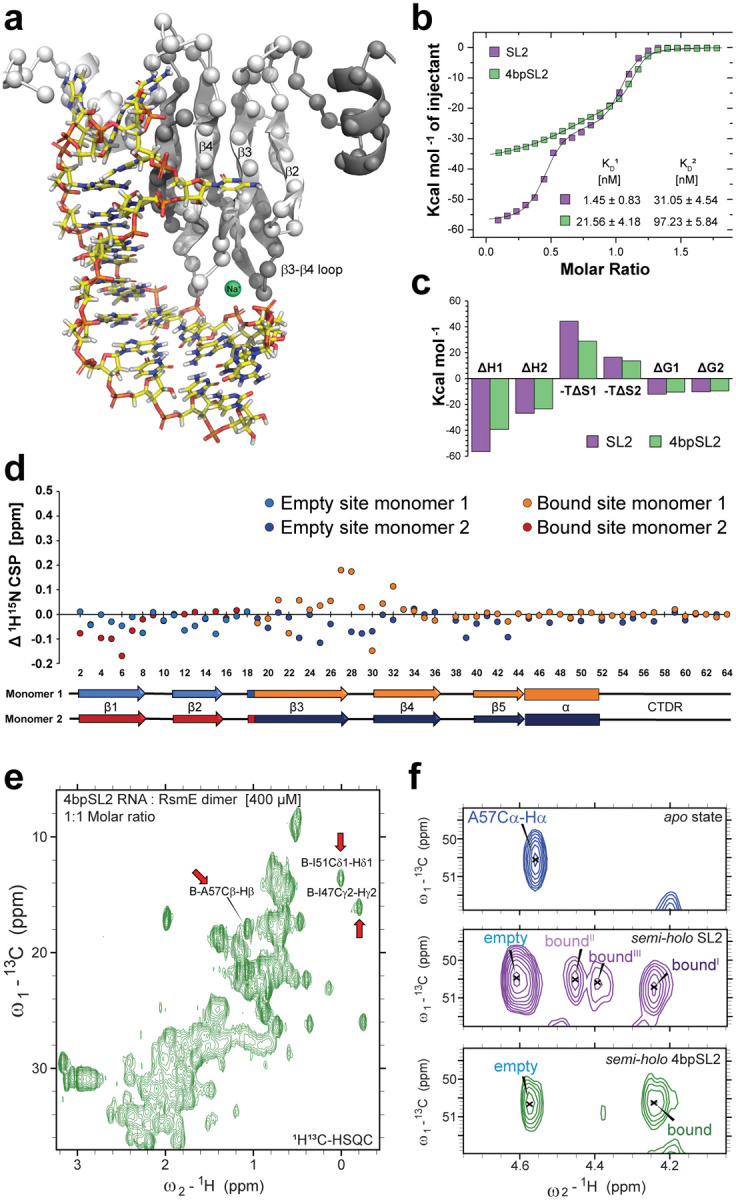
Transient interactions between the RNA stem and the empty β3-β4 loop increase binding affinity and conformational entropy in the semi-*holo* state. **a**, Semi-*holo* SL2-RsmE dimer complex from one frame of the MD simulations. Monomers are differently coloured in light and dark grey. The coordinated Sodium ion is shown in green. **b**, ITC curves for the binding of SL2 and 4bpSL2 RNA to the RsmE dimer. **c**, Bar plot with thermodynamic changes for the first and second binding events obtained from ITC. **d**, Difference in NH-CS perturbations between the semi-*holo* SL2- and 4bpSL2-RsmE dimers. **e**, Methyl region of the ^1^H^13^C-HSQC spectrum from the semi-*holo* 4bpSL2-RsmE dimer complex (green). Red arrows indicate the chemical shifts where additional minor conformation peaks were observed in the semi-*holo* SL2-RsmE dimer complex. **f**, A57 CαHα peaks in different 1H13C-HSQC spectra. From top to bottom: the *apo* state, the semi*-holo* SL2-RsmE dimer complex (purple) and the semi*-holo* 4bpSL2-RsmE dimer complex (green).

**Figure 4:**
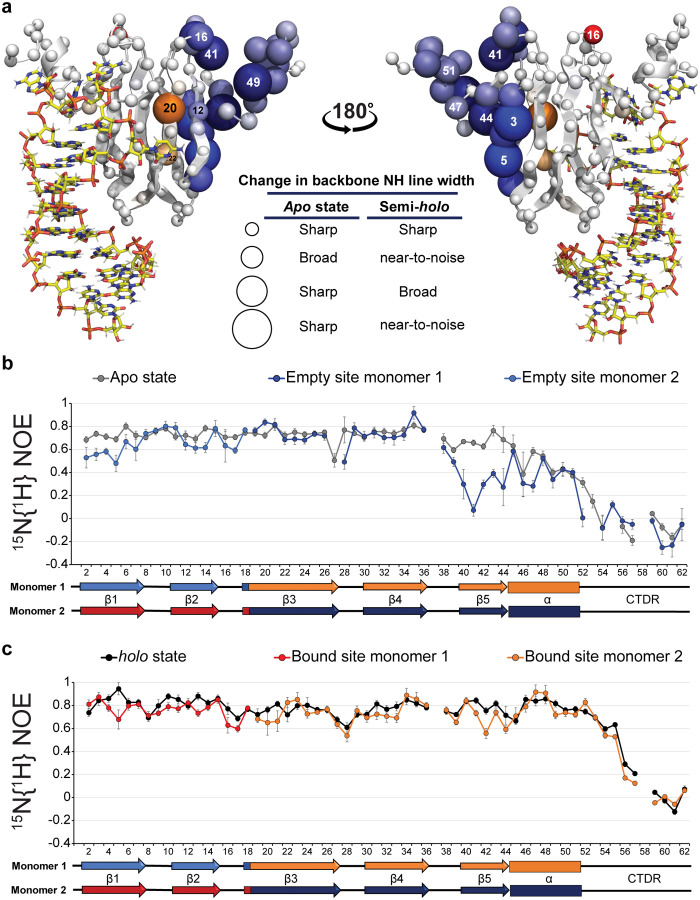
SL2 RNA binding to RsmE dimer enhances motion in its empty binding site. **a**, Mapping of line-broadening in backbone amide peaks in the semi-*holo* SL2-RsmE dimer complex. Spheres highlight the line-broadening of backbone amide peaks. The residues in the bound site are coloured in orange and red. Whereas in the empty site, they are coloured with light and dark blue. Intensity of colours and the diameter of spheres correlate with the degree of backbone amide peak line-broadening in every RsmE residue upon SL2 RNA binding. **b**, Plot of ^15^N{^1^H}-heteronuclear NOE measurements from the *apo* RsmE dimer (grey) and the empty site of the semi-*holo* SL2-RsmE dimer, as determined at 303 K. **c**, ^15^N{^1^H}-heteronuclear NOE of the *holo* SL2-RsmE dimer complex (black) and the bound site of the semi-*holo* SL2-RsmE dimer complex, as determined at 303 K.

The investigation of anti-cooperative binding in biological systems profits from the relative ease of populating and characterizing the intermediate states. From a static perspective, some crystallographic studies have shown that binding of the first ligand can deform the distant empty site, with the expected consequence of an unfavourable binding for a second ligand^12^. Conversely, other crystallographic studies on intermediate states showed no obvious structural changes on the unliganded sites that could explain the negative cooperativity^13^. NMR studies in solution have shown that negative cooperativity can indeed occur with no conformational changes bridging the two binding sites^14^. Our investigation on the intermediate states of RsmE dimer provided a mixed picture. We could explain why the binding affinity at the first binding site is stronger than at the second binding site. For the semi-*holo* state, we found multiple lines of evidence indicating that RsmE accesses more conformations in both the RNA bound site and, especially, at the empty site, compared to the *holo* forms (Fig.2 and 4). This suggests that the semi-*holo* state has higher conformational entropy compared to the *holo* state, which could contribute to the more favourable binding free energy for the first SL2 binding. In addition, the first RNA binding makes additional interactions via the stem with the β3-β4 loops in the RsmE dimer (Fig.3a), which contribute to binding enthalpy while also contributing to the large entropic penalty incurred in the canonical RNA binding site (α-helix, β1 and β5 strands). Some of this entropic penalty is offset by increased dynamics elsewhere, which is allosterically communicated to the empty site, contributing favourably to the first binding event via increased entropy. Conversely, these increased dynamics in the accessible binding site augment the entropic penalty for the second RNA binding event. The second binding event also causes subtle structural changes in the initially bound site via allostery (Fig.1b, **Supplementary Fig.6**), reducing the overall enthalpy due to less favourable interactions in the initially bound site. Altogether, from a thermodynamic point of view, our results explain well the origin of the observed difference in RNA binding affinity between the first and second binding events.

**Figure 5:**
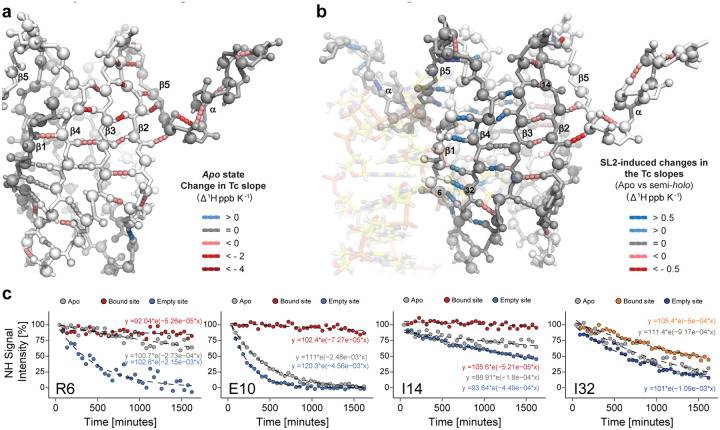
A temperature-dependent structural transition bridges structural and thermodynamic changes between the two binding sites in the RsmE dimer. **a**, Cartoon of the backbone of the *apo* RsmE dimer. Monomers are differently coloured in light and dark grey. Interrupted coloured lines correspond to H-bonds whose amide experienced non-linear temperature coefficients. Their colour-coding is indicated in the legend. **b**, Cartoon of the backbone of the semi-*holo* SL2-RsmE dimer complex. The SL2 RNA is rendered transparent to ease RsmE dimer visualization. RsmE monomers are differently coloured in light and dark grey. Residue numbers label the N atoms. Segmented coloured lines correspond to RsmE hydrogen bonds that changed their temperature coefficients upon SL2 RNA binding. **c**, Decay curves for the HDX of R6, E10, I14 and I32 backbone amides of the *apo* and semi-*holo* RsmE dimer states. They are coloured-coded using the colours in the RsmE monomer schemes in Fig.1,3 and 4. The HDX curves for other backbone amides are shown in Supplementary Fig.13.

The second and unexpected new aspect of our work relates to the allosteric pathway that couples the two binding sites. We observed no major changes in the protein dimer upon RNA binding to the first binding site with only modest CS differences between semi-*holo* and *holo* states (Fig.1c). However, close examination of the hydrogen-bond strength in the β-sheets of the RsmE dimer revealed a rigidification of the β-strands at the RNA bound site and a relaxation or weakening in the β-strands of the empty site (Fig.5b–c). This supports a model in which the backbone of the β-strands communicates an allosteric signal from the bound to the distant empty site, without major structural changes in the transition region. In the past, we^15,16^ and others^17,18^ observed that RNA binding triggered rigidification at the binding site with increased dynamics in other parts of the molecule. This can be interpreted as a conservation of conformational entropy during the binding process: the entropy loss at the RNA binding surface of the protein is compensated by increased conformational entropy at a distant site. This is what we clearly observed here as well, except that this conformational entropy compensation is now also functionally important in multivalent binding as it results in a lower binding affinity for the second RNA molecule.

Importantly, increasing the temperature — and thereby entropic effects — of the *apo* RsmE dimer in isolation led to similar relaxation of H-bonds in both binding sites (Fig.5a) in the absence of RNA binding. This observation suggests that the fold of the *apo* binding sites is inherently susceptible to subtle changes in conformational entropy. Thus, the two binding sites can be considered as two energetically frustrated structural elements^19^, where the cognate RNA binding eliminates local frustration, but enhances energetic frustration on the other binding site.

**Figure 6:**
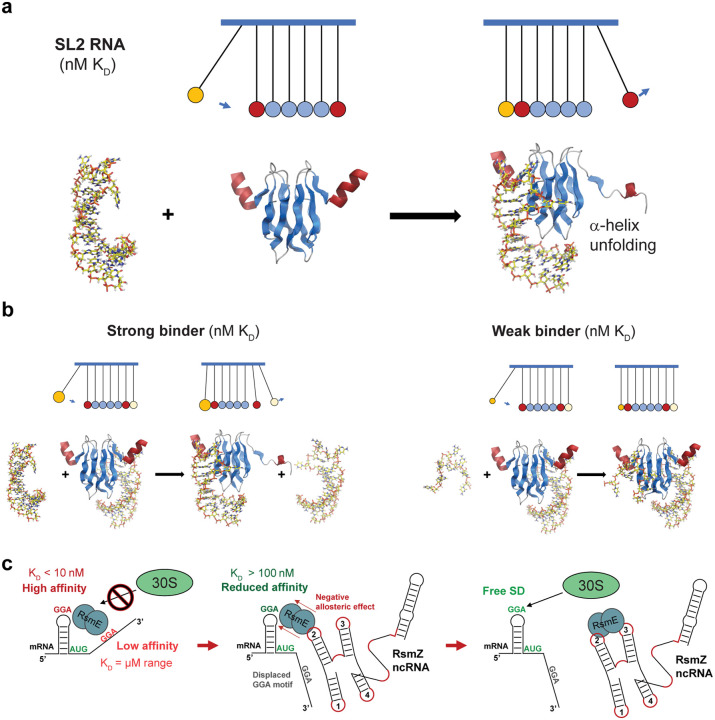
Newton’s cradle, the negative allostery in the RsmE dimer and the de-repression of mRNA translation in bacteria. **a**, Newton’s cradle analogy for the binding of SL2 RNA to RsmE dimer and the emergence of a high energy state on the empty binding site. **b**, Newton’s cradle analogy for the understanding of the affinity-associated allostery. **c**, The biological function of the negative allostery in the RsmE dimer.

In the RsmE dimer, we observe how conformational entropy apparently propagates through a network of β-strands connecting the two RNA-binding sites, creating a dynamic coupling analogous to Newton’s cradle (Fig.6a). Entropy-driven allostery mechanisms^20^ have been observed in various systems, including Newton’s cradle-like dynamics in bimetallic porphyrin complexes^21^. Similarly, entropy redistribution enables allosteric communication in metalloregulatory proteins, akin to the energy propagation in Newton’s cradle^22^. In T4 lysozyme, cooperative allosteric transitions rely on concerted motions within a flexible transmission network^23^, whereas in the murine urokinase-type plasminogen activator, a disordered light chain enhances protease activity by transmitting allosteric effects through β-strands, altering distant loop dynamics and catalytic activity similar to Newton’s cradle-like propagation^24^. In a similar manner, allostery is mediated across domains by subtle rearrangements of conformational sampling in β-sheets of the PPIase^25^ and WW domains^26^ in Pin1 and PDZ domains^27^. McLeish et al. proposed that fluctuation-driven allostery can be conceptualized through emergent collective motions^28^. They show how coupling of these collective motions between the two homotypic monomers of catabolite activator can lead to the observed strong negative allostery of cAMP binding. They predict enhanced dynamics at the non-bound site, which is quenched by the second binding event, consistent with the behavior of the RsmE dimer. Kornev^29^ proposed that entropy-driven allosteric communication arises from the emergent behavior of self-organized oscillators, forming semi-rigid clusters that transmit signals efficiently without necessitating structural rearrangements. Our findings suggest that the RNA binding mechanism of the RsmE dimer leverages a similar entropy-driven process to achieve functional coordination, where the β-strand network acts as a rigid conduit for transmitting conformational entropy, enabling functional communication across distant sites.

In the Newton’s cradle analogy, the binding RNA stem-loop can be seen as the incoming ball, while the β-strands of the RsmE dimer represent the middle balls. In an ideal Newton’s cradle, the kinetic energy imparted to the outer ball is transferred through the middle balls without apparent displacement, and ultimately to the ball on the opposite side, which then moves away, but can rebind. By analogy, in our system, the kinetic energy may be replaced by conformational entropy that is transmitted via the β-strands leading to unfolding of the helix on the other side (Fig.6a). The analogy with the cradle also comes from the in-line compression and expansion that is transmitted by the hydrogen-bond network. The cradle analogy furnishes a rationale for why we cannot detect anticooperativity with weak RNA binding^7^: weak binding results in insufficient conformational entropy transfer to destabilize the distant binding site, and therefore fails to weaken the affinity for a second RNA (Fig.6b). Similarly, if a second RNA binds to the second site with very high affinity, the process can reverse causing increased entropy at the initially bound site, which can lead to lower affinity and partial dissociation of the initially bound RNA on the other side. This lowered affinity at the second binding site is functionally important as it allows us now to explain how the RsmZ ncRNA can displace tightly bound RsmE dimers from the bound mRNAs. In light of our results, we can envisage that this happens in two steps (Fig.6c). In a first step, SL2 of the ncRNA RsmZ displaces the weakly bound mRNA because of the high affinity of SL2 for binding RsmE. SL2 binding then allosterically triggers increased motion in the second RNA binding site that is bound by the SD stem-loop and therefore entropically destabilizes it. RsmZ SL3, which is in cis with SL2, can then compete with and displace the SD sequence, resulting in the detachment of the RsmE dimers from the mRNAs and leading to mRNA de-repression and ultimately gene expression. We anticipate that similar mechanisms may facilitate the displacement of multiple RNAs in other multivalent RNA binding proteins.

In general terms, the negative cooperativity observed in the RsmE dimer illustrates, mechanistically, how evolution can fine-tune protein folds and frustrated binding sites to thermodynamically couple distant allosteric and functional sites. This thermodynamic coupling can regulate, in an energetically efficient manner, diverse biological process, including cAMP sensing and the associated gene expression in bacteria^14,30,31^; calmodulin function^32^, cell growth and proliferation via PDZ domains in mammals^33^; biochemical processes such as enzyme catalysis in monomeric proteins^34^, communication in homodimeric and multidomain proteins^35,36^, and even pathological processes such as the toxicity of prion proteins^37^.

## Methods:

### Expression and purification of the RsmE dimer

The coding sequence of RsmE dimer was cloned in pET28b plasmid (Novagen), which added a 6X-His tag to the C-terminal end of RsmE. To ease the quantification of the RsmE dimer molar concentration, a Proline-to-Tryptophan (P64W) mutation was introduced at the C-terminal end of RsmE to increase the 280nm UV absorbance of the RsmE dimer. Plasmid DNA sequence was verified using Sanger sequencing and BL21-Codon Plus (DE3)-RIL *E. coli* cells (Agilent Technologies) were transformed with pET28b-64WRsmE for protein expression. Once the bacterial culture reached an OD600 of 0.6–0.8, expression was induced with 1 mM isopropyl-β-d-thiogalactopyransoide (IPTG) and carried out for 4 hours at 37 °C. For isotopic labelling of RsmE dimer, *E. coli* cells were grown in M9-minimal medium containing 99%−^15^NH_4_Cl and either D-glucose or 99%−^13^C_6_-D-glucose to express either uniformly ^15^N-labelled RsmE dimer or ^13^C^15^N-labelled RsmE dimer. For the perdeuterated RsmE dimer sample, the M9 medium was prepared in 100 % D_2_O, instead of H_2_O. *E. coli* cells were pelleted by centrifugation and resuspended in a lysis buffer: 50 mM potassium phosphate, pH 8, 300 mM NaCl, 10 mM imidazole and protease inhibitor cocktail (cOmplete EDTA-free, Roche). The cells were lysed using a microfluidizer (Microfluidics) and the clarified lysates containing RsmE dimer were loaded on Ni-NTA-agarose beads (Qiagen). The Ni-bound RsmE dimers were washed with a solution containing 50 mM potassium phosphate, 1 M NaCl, 10 mM imidazole, pH 8, followed by stepwise washes with increasing concentration of imidazole (30 and 100 mM) and RsmE dimers were eluted with 250 mM imidazole. Proteins were dialysed overnight against NMR buffer (30 mM NaCl, 50 mM Potassium phosphate, pH 7.2) and concentrated with a centricon (3 kDa molecular mass cut-off membrane, Vivascience) to reach the desired protein concentration. Samples were tested for purity by SDS-PAGE (Laemmli, 1970). Protein concentrations were determined by their 280 nm UV absorption and the theoretical Molar extinction coefficient of the RsmE dimer (6990 M^−1^ cm^−1^) was calculated using the ExPASy tool ProtParam^38^.

### RNA in vitro transcription and purification

SL2 RNA (5´-GGGCCATCAAGGACGATGGUCC-3´) and 4bpSL2 RNA (5´-GGATCAAGGACGATCC −3´) were in-vitro transcribed from double stranded DNA templates (Microsynth) using in house-produced T7 RNA polymerase and unlabelled triphosphate nucleotides for transcription^39^. The products were purified by anion exchange chromatography under denaturing conditions, followed by butanol extraction as previously described^40^. Pelleted RNAs were resuspended in water, incubated at 98°C for one minute and slowly cooled for refolding. RNA purity was tested using urea-PAGE. Concentrations were determined using the 260 nm UV absorbance. Their theoretical molar extinction coefficient (SL2: 243.14 mM^−1^ cm^−1^ and 4bpSL2: 183.1 mM^−1^ cm^−1^) and final concentration were calculated using the online-tool RNACalc^41^.

### NMR spectroscopy and NMR data analysis

NMR experiments were recorded at 313 K for the apo ^15^N or ^13^C^15^N-RsmE dimers and all other RNA-RsmE dimer complexes on Bruker Avance III 500, 600, 700 or 900 MHz spectrometers. All spectrometers were equipped with cryoprobes. All samples were prepared in NMR buffer (30 mM NaCl, 50 mM Potassium phosphate, pH 7.2) with 3 % D2O. Complete assignments of the ^1^H^15^N-HSQC spectra for the apo RsmE dimer and SL2 RNA bound RsmE dimer, were obtained with triple resonance spectra (HNCACB, HNCA, HN(CA)CO and HNCO)^42^. To assign ^1^H^13^C chemical shifts of the amino acid side chains, 3D HcCH Total correlation spectroscopy (TOCSY) and 3D HCH-Nuclear Overhauser effect spectroscopy (NOESY) experiments were recorded. RsmE-bound SL2 RNA chemical shifts were retrieved from the PDB entry “2mfe” of the solution structure of RsmE dimer bound to two SL2 RNAs. For ^1^H^13^C-HSQC, HcCH-TOCSY, HCH-NOESY and filtered-edited NOESY experiments, protein or RNA-protein samples were lyophilized overnight and resuspended under low humidity conditions in 100 % D2O. For ^15^N{^1^H}-heteronuclear NOE experiments, perdeuterated ^15^N-RsmE dimer samples were prepared, and spectra were recorded at 303K. ^1^H irradiation was applied for the last 3s of a 5s recycle delay and was omitted in the reference experiment. NMR data were processed with Topspin 3.0 software. NMR spectra were analysed in NMRFAM-SPARKY software^43^. Backbone amide chemical shift perturbations (Δδ_NH_) were calculated with the following equation:

$$\Delta \delta_{NH}=\sqrt{\Delta {\delta_{H}}^{2}+0.153\Delta {\delta_{N}}^{2}}$$

where Δδ_H_ and Δδ_N_ are the ^1^H and ^15^N chemical shift difference between the compared states.

For the calculation of random coil chemical shifts of the apo RsmE amino acid sequence at 313K and pH 7.2, the chemical shifts and the sequence correction factors were derived as reported by Kjaergaard & Poulsen, 2011^44^. The basis to determine the sequence correction factors were described in Schwarzinger et al., 2001^45^. The glycine correction factors and the temperature coefficients were derived from Kjaergaard et al., 2011^46^.

### Determination of amide proton temperature dependence

^1^H^15^N-HSQC experiments from apo RsmE dimer and SL2 bound RsmE dimer were acquired at 283, 288, 293, 298, 303, 308, 313, 318, 323 and 333 K. After heating to 333 K, thermal reversibility was checked by returning to 298 K. The apo RsmE dimer and SL2 RNA-bound RsmE dimer spectra were completely reversible. A sample of 1mM Sodium 3-(trimethylsilyl)propane-1-sulfonate (DSS) in NMR buffer (30 mM NaCl, 50 mM Potassium phosphate, pH 7.2) with 3 % D_2_O was used to reference the ^1^H chemical shifts at different temperatures. Chemical shift changes were monitored as a function of the temperature and fitted to a linear regression. The temperature coefficients were obtained from the slope, in ppb K^−1^. NMR assignment of ^1^H^15^N-HSQC spectra at different temperatures was conducted by manually tracking the systematic shifts. The assigned ^1^H^15^N-HSQC spectrum at 313K was used as a reference.

### Hydrogen-deuterium exchange

A series of ^1^H^15^N-HSQC experiments were recorded at 313K after dissolving lyophilized RsmE dimer samples in D_2_O. Exchange rates were determined from time dependence of peak intensities. The decay curves were fitted to a first-order rate:

h(t)=Aexp(−kt)

Where *h(t)* is NH-CS peak height at time t, A is fitted to the initial intensity, and k is the exchange rate. The best fit for the exchange rate *k* is reported.

### Isothermal titration calorimetry (ITC)

Prior to ITC experiments, purified RsmE dimer and RNA samples were dialyzed against the same NMR buffer (buffer 30 mM NaCl, 50 mM Potassium phosphate, pH 7.2) and the calorimeter was calibrated following manufacturer’s instructions. All ITC experiments were performed on a VP-ITC instrument from MicroCal, where RNA was titrated into the RsmE protein. RsmE monomer concentration in the cell (cell volume = 1.4644 mL) was calculated to be 10 μM. The syringe was loaded with 280 μL of RNA containing solution (80–100 μM concentration). Experiments were conducted twice at 298 K, typically consisting of 40–70 injections of 4–10 μl. The injection speed was 2 s/μl, with 5 min interval between injections. The syringe stirred at a rate of 307 rpm. Data were integrated and fitted to a sequential binding model using Origin 7.0 software. The dissociation constants (K_d_) and the enthalpic terms (ΔH) were derived directly from the fitted model. The entropic terms (-TΔS) were calculated using the standard Gibbs’ Free energy equation (Δ*G* = Δ*H* − *T*Δ*S*), where ΔG was calculated from the following equation:

$$\Delta G=-RTlnK_{d}$$

The mean and standard deviation of the K_d_ were calculated from three ITC replicates, as well as the mean values for ΔH, −TΔS and ΔG.

### Molecular Dynamics Simulations

Conformer 13 of the NMR solution structure bundle of RsmE dimer bound to two SL2 RNA (PDB: 2mfe) was used as the starting structure in all MD simulations. None, one or two SL2 RNA were removed prior to solvation (explicit-solvent conditions) and equilibration of the different systems. Three or four simulations were performed for each system. All simulations ran for the minimal length of 10 μs. The ff14SB and bsc0χOL3 force fields were implemented to describe protein and RNA potentials, respectively^47,48^. The simulations were performed using AMBER18 software. Standard protocols for equilibration and simulation of protein/RNA complexes were applied^49^. All atomistic distances were calculated from the trajectories using the cpptraj module in AMBER18. Further information on the analysis of fractional sodium occupancy in MD simulations can be found in Supplementary Text 3.

## Extended Data

**Extended data 1:**
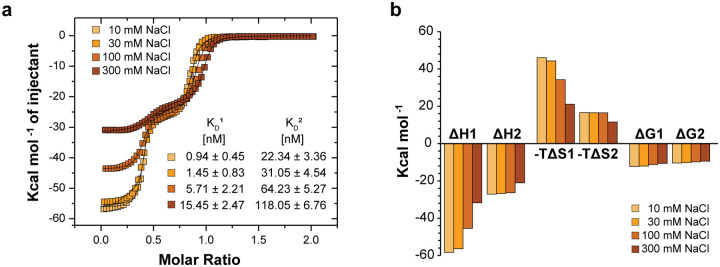
Increased ionic strength strongly reduces the binding affinity of first bound SL2 RNA for RsmE dimer. **a**, Overlaid ITC curves for the binding of SL2 RNA to the RsmE dimer with increasing concentration of NaCl in the buffer (50 mM Potassium phosphate, pH 7.2). **b**, Bar plot with thermodynamic changes for the first and second binding events. Values can be found in Supplementary Table 1.
